# Gender as a moderating variable in the relationship between childhood traumatic experiences and guilt in adolescents

**DOI:** 10.3389/fpsyg.2026.1873429

**Published:** 2026-09-18

**Authors:** Marjorie Reyna-Tudor, Diego Aranda-Rodriguez, Carlos Pérez-Lara

**Affiliations:** School of Psychology, Faculty of Health Sciences, Universidad César Vallejo, Trujillo, La Libertad, Peru

**Keywords:** adolescents, childhood traumatic experiences, gender moderation, guilt, Latin America, psychological distress, self-conscious emotions, trauma exposure

## Abstract

Childhood traumatic experiences are associated with the development of self-conscious emotions such as guilt during adolescence; however, the role of gender in this relationship remains insufficiently understood, particularly in Latin American contexts. This study aimed to examine whether gender moderates the relationship between childhood traumatic experiences and guilt in adolescents in Trujillo, Peru. A cross-sectional design was employed with a non-probabilistic sample of adolescents (*N* = 200). Childhood trauma was assessed using the Child and Adolescent Trauma Screen, and guilt was measured using the Guilt and Shame Experience Scale (GSES). Moderation analysis was conducted to examine the interaction between trauma and gender. The results indicated that childhood traumatic experiences were significantly associated with guilt. Gender also showed a significant effect and moderated this relationship. Specifically, the association between trauma and guilt was significant among male adolescents but not among females, indicating gender-based differences in trauma-related emotional processing. These findings highlight the relevance of gender in understanding the relationship between childhood trauma and guilt during adolescence. The study provides evidence from a Latin American context and underscores the need for gender-sensitive approaches in the assessment and intervention of trauma-related emotional outcomes, supporting the development of targeted prevention and intervention strategies aimed at reducing psychological distress in adolescents.

## 1 Introduction

Guilt is a subjective emotional experience associated with the perception of having committed an error, offense, or transgression, and is typically accompanied by self-criticism and psychological distress (Descriptores en Ciencias de la Salud, 2026). In the context of traumatic experiences, guilt may also emerge through victim-blaming processes, in which responsibility is incorrectly attributed to the victim, either by others or by the individual themselves (Houdek et al., 2025). This phenomenon has important implications for adolescent mental health, particularly when it arises as a response to adverse developmental experiences.

Epidemiological evidence highlights both the prevalence of childhood trauma and its emotional consequences. The World Health Organization estimates that 1 in 5 women and 1 in 7 men have experienced sexual abuse during childhood (OMS, 2024). Similarly, global data indicate that between 1 and 53% of girls and 4 and 60% of boys are exposed to sexual abuse before the age of 18, with more than 55% reporting associated feelings of guilt (Melville et al., 2014). In the Peruvian context, during the COVID-19 pandemic, 59.3% of adolescents reported physical abuse and 40.7% psychological violence from caregivers, both of which were associated with the emergence of guilt (Ministerio de Salud, 2021). More broadly, over 80% of individuals exposed to traumatic events report experiencing trauma-related guilt at some point in their lives (Davis et al., 2023). Together, these findings underscore the relevance of guilt as a frequent emotional response following exposure to adversity.

From a public health perspective, childhood traumatic experiences represent a major risk factor for the development of mental health disorders across the lifespan. In line with Sustainable Development Goal 3 (Good Health and Well-being), particularly target 3.d, which emphasizes risk reduction and the strengthening of early response systems (Organización de las Naciones Unidas, 2022), understanding the psychological mechanisms associated with trauma is essential. In this context, examining the role of gender in shaping the relationship between traumatic experiences and guilt may contribute to improving early detection, guiding mental health screening, and informing the development of targeted interventions in school and community settings.

Evidence from different regions supports the association between childhood trauma and guilt-related processes. In Asia, specifically in China, families with two children under 18 years of age (46.70%) have been associated with childhood trauma experiences such as emotional or physical neglect, with victims frequently reporting feelings of guilt, particularly in contrast to single-child families (Tong et al., 2022). In Europe, research conducted in Spain has shown that childhood sexual abuse and neglect are associated with increased attribution of blame toward the family environment, while factors such as the relationship with the perpetrator and the nature of the abuse contribute to both self-blame and external blame processes (Cantón-Cortés and Rosario Cortés, 2015).

In North America, a study conducted in the United States reported that 37% of adolescents had experienced social bullying, 30% physical bullying, and more than half verbal bullying, with 50% attributing responsibility to the victim and 27% believing that the victim had “brought it upon themselves” (Chapin and Coleman, 2017). Similarly, in the state of Ohio, 83% of adolescents presented at least one traumatic symptom associated with sexual abuse, and those who engaged in self-blame exhibited higher levels of symptoms such as anxiety, fear, and nightmares. Notably, the likelihood of self-blame doubled when parents did not believe the disclosure of abuse (Melville et al., 2014). Within this context, several studies indicate that the experience of guilt is not homogeneous across genders. Women tend to report higher levels of guilt than men, which has been linked to socialization processes and greater interpersonal sensitivity (Roos et al., 2014). Likewise, differences have been observed in the propensity to experience shame and guilt, with women generally showing higher levels, and with shame being positively associated with internalized anger in both sexes (Lutwak et al., 2001). However, these differences should be interpreted with caution, as gender-based stereotypes may overestimate female emotionality, and observed variations are typically moderate and influenced by contextual, cultural, and measurement-related factors (Else-Quest et al., 2012).

In Latin America, evidence also highlights the impact of childhood trauma on emotional outcomes. In Venezuela, child maltreatment within the household has been associated with significant physical and emotional consequences, including prevalence rates of 34.54% for physical abuse, 30% for psychological abuse, 26.36% for neglect, and 9.09% for sexual abuse. Although guilt is not explicitly assessed, outcomes such as low self-esteem, depression, anxiety, post-traumatic stress disorder, and dissociative symptoms have been linked in the literature to the internalization of guilt (Fernández et al., 2020). In Colombia, research involving survivors of armed conflict in Medellín identified internal guilt, expressed through personal questioning about the traumatic event, as well as displaced guilt, reflected in the justification of the perpetrator’s actions (Bedoya-Olaya et al., 2023).

Within this regional context, data from Peru indicate that the Women’s Emergency Centers reported 25,067 cases of sexual violence between January and October 2024, of which 70.25% involved minors; similar figures were reported in the previous year, with 30,387 cases and 72.16% involving minors. These experiences have been associated with feelings of guilt and confusion, difficulties in verbalizing traumatic events, a predisposition to substance use, and limitations in emotional development, all of which increase the risk of mental health problems (Ministerio de la Mujer y Poblaciones Vulnerables, 2024). Additionally, in Huánuco, testimonies from girls and adolescents who were victims of sexual violence revealed the presence of guilt, silence, fear, and revictimization, although professional, familial, and social support were identified as protective factors that facilitate recovery processes (Save the Children International, 2019). Furthermore, in the La Libertad region, specifically in the province of Chepén, childhood traumatic experiences were reported at predominantly moderate levels in a school population, with notable rates of physical abuse (46%), emotional abuse (44%), emotional neglect (53%), and physical neglect (44%), while sexual abuse reached 15% at a mild level (Aguilar and Gonzales, 2024).

A growing body of research indicates that exposure to childhood traumatic experiences is associated with the development of negative self-conscious emotions, particularly guilt and shame, during adolescence, as well as with an increased risk of mental health problems. The accumulation of traumatic events has been consistently linked to higher levels of guilt, shame, and psychological distress (Cândea and Szentagotai-Tătar, 2018; Stotz et al., 2015; Kletter et al., 2009), which in turn are associated with conditions such as depression, anxiety, and trauma-related disorders (Valladares-Garrido et al., 2023). Within this framework, gender has been identified as a relevant factor influencing how these emotional responses are experienced and expressed, with evidence suggesting that it may act as a moderating variable in the relationship between traumatic experiences and mental health outcomes (Scharpf et al., 2025; Roman-Lazarte et al., 2023; Baños-Chaparro, 2023). In addition, guilt has been associated with increased psychological distress among adolescents exposed to adverse experiences (Arcos Naquira, 2019). Together, these findings highlight the importance of examining the interaction between childhood trauma, guilt, and gender in adolescent emotional development.

Childhood traumatic experiences refer to exposure to potentially traumatic events such as abuse, neglect, violence, or disasters, as assessed by the Child and Adolescent Trauma Screen (CATS), which is based on DSM-5 criteria for post-traumatic stress disorder (Sachser et al., 2014). According to the DSM-5, trauma involves direct experience, witnessing, or learning about events related to death, serious injury, or sexual violence (American Psychiatric Association, 2014). Exposure to such events during childhood has been associated with disruptions in emotional, cognitive, and social development (Cruz et al., 2022). From a theoretical perspective, developmental trauma theory suggests that chronic exposure to adverse experiences interferes with neurological and psychosocial functioning (Gregorowski and Seedat, 2013), while complex trauma theory proposes that repeated exposure may lead to long-term alterations in identity formation and emotional regulation (Herman, 1992, as cited in Rushforth et al., 2022). Together, these frameworks highlight the pervasive impact of trauma across multiple domains of development.

In the present study, childhood traumatic experiences were operationalized as the cumulative number of potentially traumatic experiences reported by each participant, based on the exposure component of the CATS. This approach allowed the study to examine whether the number of reported traumatic experiences was associated with guilt and whether this association varied according to gender, also the dimensions: exposure to traumatic events, post-traumatic stress symptoms (intrusions, avoidance, cognitive and affective alterations, and hyperarousal), and functional impairment across academic, social, family, and emotional domains (Berliner et al., 2021). This multidimensional approach allows for a comprehensive assessment of trauma by capturing both exposure and its consequences. In addition, trauma outcomes are influenced by contextual factors, such as family and community environments, as well as individual resources, including resilience (Yearwood et al., 2021; Burga-Cachay et al., 2023; Sölva et al., 2023).

Guilt is conceptualized as a self-conscious emotion involving the negative evaluation of one’s behavior and the perception of personal responsibility for a negative outcome (Malinakova et al., 2020). From an attributional perspective, guilt is particularly relevant when individuals interpret an adverse event in relation to their own actions or perceived responsibility. Attribution theory proposes that individuals evaluate the causes of events and may attribute responsibility to themselves or to external circumstances (Weiner, 1985). Similarly, Janoff-Bulman's (1979) distinction between behavioral and characterological self-blame suggests that interpretations involving personal responsibility may contribute to different forms of self-evaluation following adverse experiences. Thus, exposure to childhood trauma may be followed not only by psychological distress but also by cognitive processes through which adolescents attempt to understand what happened and determine whether they were responsible.

These attributional processes can be further understood through the transactional model of stress and coping proposed by Lazarus and Folkman (1984). From this perspective, stressful or traumatic events do not determine emotional responses directly; rather, responses are influenced by how individuals appraise the event and their perceived ability to cope with it. In the context of childhood trauma, adolescents may retrospectively evaluate adverse experiences and interpret them in terms of responsibility, controllability, or what they believe they could have done differently. When such appraisals involve perceived personal responsibility, they may contribute to guilt. Therefore, cognitive appraisal provides a conceptual link between exposure to traumatic experiences and the development or expression of guilt.

The framework of self-conscious emotions provides a complementary explanation for this process. Self-conscious emotions emerge when individuals evaluate themselves in relation to personal standards, social expectations, or perceived responsibility (Tangney and Dearing, 2002). Within this framework, guilt is generally related to evaluations of specific behaviors and perceived responsibility, whereas shame involves broader negative evaluations of the self. In adolescence, increasing self-reflection and sensitivity to social norms may make these evaluative processes particularly relevant when adolescents interpret adverse experiences (González et al., 2019; Malinakova et al., 2020). Accordingly, childhood trauma may be associated with guilt when adolescents interpret the experience through processes of responsibility attribution and self-evaluation.

However, these cognitive and emotional processes occur within a social context. Gender can be understood as a sociocultural construct involving socially assigned roles, expectations, and meanings that may influence emotional expression and interpersonal behavior (Ministerio de la Mujer y Poblaciones Vulnerables, 2017). Social role theory (Eagly, 1987) proposes that gendered expectations are learned through socialization and may shape how individuals behave and respond emotionally to social situations. Consequently, adolescents may differ in how they interpret, regulate, and express emotions such as guilt depending on the gender-related expectations present in their social environments.

Taken together, these perspectives provide an integrated conceptual pathway for the present study. Childhood traumatic experiences constitute adverse events that may require cognitive appraisal; these appraisals may influence how adolescents attribute responsibility for what occurred; and responsibility-related appraisals may contribute to guilt as a self-conscious emotion. At the same time, gender-related socialization and expectations may shape the way these appraisals and emotional responses are experienced or expressed. Therefore, gender may influence the strength of the association between childhood traumatic experiences and guilt, rather than simply determining the overall level of guilt. This provides the conceptual rationale for examining gender as a moderator of the relationship between childhood traumatic experiences and guilt.

The moderation model was examined empirically using an interaction between childhood traumatic experiences and gender. This analytical approach allows assessment of whether the strength and direction of the association between childhood traumatic experiences and guilt vary across gender groups.

Despite growing evidence, there is limited research examining gender as a moderating variable in the relationship between childhood trauma and guilt, particularly in Latin American adolescent populations. Most studies have been conducted in high-income contexts, limiting the understanding of sociocultural influences on emotional processing. Addressing this gap may contribute to the development of culturally sensitive and gender-responsive mental health strategies.

Accordingly, the present study aims to determine whether gender moderates the relationship between childhood traumatic experiences and guilt in adolescents in Trujillo, Peru.

## 2 Method

### 2.1 Design

According to OECD and Eurostat (2018), the present research was of a basic type. This study employed a quantitative (Ñaupas et al., 2018), cross-sectional, non-experimental design and a non-probabilistic convenience sampling. A regression-based moderation analysis framework was used to examine whether the association between childhood traumatic experiences (independent variable) and guilt (dependent variable) varied as a function of gender (moderator) (Hayes, 2018). Given that the variables were not manipulated, the study relied on observational data to analyze naturally occurring relationships. The analytical approach was theory-driven, aiming to assess the conditional effect of childhood trauma on guilt across gender groups, thereby contributing to the understanding of variability in emotional responses during adolescence (Hernández-Sampieri and Mendoza, 2014).

### 2.2 Participants

A total of 220 adolescents were initially assessed using a brief checklist-type questionnaire developed for the initial screening process. This questionnaire was used to collect sociodemographic information, verify the predefined inclusion and exclusion criteria, and identify whether adolescents had experienced at least one potentially traumatic childhood experience. Adolescents who did not report any potentially traumatic childhood experience were not included in the study sample. Importantly, the CATS was not administered at this initial screening stage and no CATS cutoff score was used as an eligibility criterion. Of the 220 adolescents initially assessed, 20 were excluded. Specifically, 16 participants were excluded because they left items unanswered in the questionnaire, while 4 explicitly refused to participate in the study. Consequently, the final analytical sample consisted of 200 adolescents (100 males and 100 females) aged 15–16 years. All questionnaires included in the final analytical dataset were complete; therefore, no missing data were present in the final analyses.

The inclusion criteria were being 15 or 16 years old, belonging to the same socioeconomic group, having resided in Trujillo for more than 5 years, living with at least one parent, and reporting at least one potentially traumatic childhood experience during the initial screening. The exclusion criteria were having an intellectual or sensory disability that could limit autonomous participation, insufficient command of the language used in the assessment, a diagnosis of a neurological or psychiatric disorder, receiving or having received formal psychological or psychiatric treatment during the previous 6 months, completing the questionnaire incompletely, or explicitly refusing to participate in the study.

The sample size was determined *a priori* using G*Power software (version 3.1.9.7), assuming a medium effect size (f^2^ = 0.15), a significance level of α = 0.05, statistical power (1 – β) = 0.95, and two predictors. These parameters were selected to ensure sufficient sensitivity to detect the expected effects while minimizing the probability of Type I and Type II errors (Cárdenas and Arancibia, 2016). Participation was voluntary, and no financial or material incentives were provided.

Although participants with known neurological or psychiatric diagnoses, intellectual or sensory disabilities that could limit autonomous participation, and recent psychological or psychiatric treatment were excluded, no comprehensive clinical or neurodevelopmental assessment was conducted as part of the study. Therefore, the absence of a reported diagnosis should not be interpreted as evidence that participants were free from undiagnosed psychological, psychiatric, or neurodevelopmental conditions. These characteristics were not included as covariates in the statistical analyses and are therefore considered when interpreting the findings.

### 2.3 Instruments

Childhood traumatic experiences were measured using the Child and Adolescent Trauma Screen (CATS), which consists of 40 items distributed across three domains: 15 items assessing exposure to traumatic events, 20 items evaluating post-traumatic stress symptoms, and 5 items measuring psychosocial functioning. The CATS includes a 15-item checklist assessing exposure to potentially traumatic events, covering different types of traumatic experiences, including accidents or serious injuries, natural disasters, violence within the family or community, sexual abuse, traumatic loss, serious or distressing medical experiences, and other potentially traumatic events. In the present study, the exposure component of the CATS was used to quantify cumulative trauma exposure based on the number of traumatic experiences reported by each participant. Therefore, participants could report more than one type of traumatic experience, and the resulting score reflected the total number of endorsed traumatic experiences. The instrument has shown reliability ranging from good to excellent, with Cronbach’s alpha coefficients between 0.88 and 0.94. Evidence of convergent validity has been reported through moderate to strong correlations with measures of depression (*r* = 0.62–0.82) and anxiety (*r* = 0.40–0.77), while discriminant validity has been supported by low to moderate correlations with externalizing symptoms (*r* = −0.15–0.43). Furthermore, confirmatory factor analysis has supported the DSM-5-based structure of the instrument, reflecting four symptom dimensions: re-experiencing, avoidance, and negative alterations in mood and cognition. In the present study, childhood traumatic experiences were operationalized as the cumulative number of potentially traumatic experiences reported by each participant. The primary analyses therefore focused on cumulative trauma exposure rather than on the separate effects of individual trauma types. For this study, the instrument was also subjected to cultural adaptation through 3 expert judgment, obtaining an Aiken’s *V* coefficient of 1.00 for relevance, pertinence, and clarity, with a 95% confidence interval.

Guilt was assessed using the Guilt and Shame Experience Scale (GSES), a self-report questionnaire composed of 8 items designed to measure experiences of guilt and shame (Malinakova et al., 2020). The scale has demonstrated adequate psychometric properties. Internal consistency for the total scale yielded a Cronbach’s alpha of 0.89 (95% CI: 0.89–0.89), while the subscales showed alpha coefficients of 0.80 (95% CI: 0.79–0.80) for shame and 0.83 (95% CI: 0.83–0.84) for guilt. Additionally, McDonald’s omega coefficient for the total scale was 0.92, indicating high internal reliability. For the purposes of this study, the instrument was culturally adapted to the Peruvian context through 3 expert judgment involving three specialists. Content validity was assessed using Aiken’s *V*, yielding a value of 1.00 for relevance, pertinence, and clarity, with a 95% confidence interval.

### 2.4 Procedures

Prior to data collection, authorization was obtained from the administrative authorities of the educational institution. Subsequently, coordination was established with classroom teachers to schedule the administration of the assessment instruments. In the initial phase, informed consent forms were distributed to parents or legal guardians through the students’ school agendas. Only adolescents whose guardians provided signed consent were invited to participate in the study. Participants were recruited from the participating educational institution according to predefined eligibility criteria. Eligible participants were adolescents aged 15–16 years who had lived in Trujillo for more than 5 years, belonged to the socioeconomic group defined for the study, and lived with at least one parent. Participants were excluded if they had an intellectual or sensory disability that limited autonomous participation, insufficient command of the study language, a diagnosed neurological or psychiatric disorder, formal psychological or psychiatric treatment during the previous 6 months, incomplete questionnaires, or an explicit refusal to participate. Recruitment was conducted using non-probabilistic sampling based on the predefined eligibility criteria. No financial or material incentives were provided. In addition, informed assent was obtained directly from the adolescents prior to data collection. Data collection was conducted collectively in classroom settings under standardized conditions. The questionnaires were self-administered by the participants, while trained members of the research team provided standardized instructions regarding the purpose of the study and the procedure for completing the instruments. Approximately 20 min were provided for questionnaire completion. Researchers remained available throughout the administration to clarify questions concerning the instructions or completion procedure, without influencing participants’ responses. Participation was voluntary, and adolescents were informed that they could decline participation. Confidentiality of the information provided was emphasized, and the data were handled in accordance with the ethical procedures approved for the study. Upon completion, the questionnaires were collected by the research team and participants were thanked for their participation.

### 2.5 Statistical analysis

Data were analyzed using IBM SPSS Statistics (version 30) and the PROCESS macro (Hayes, 2018). The analytical strategy consisted of descriptive and inferential stages. Descriptive statistics included frequencies, means, and standard deviations. The distribution of the data was assessed using the Kolmogorov–Smirnov test with Lilliefors correction, given that the sample size exceeded 50 participants. Based on the normality assessment, parametric procedures were used where appropriate, including independent-samples t-tests for comparisons between groups, while associations between variables were analyzed using Spearman’s rho.

To test the study hypothesis, a moderation analysis was performed using multiple linear regression within the PROCESS macro (Model 1). Gender was specified as the moderating variable in the relationship between childhood traumatic experiences and guilt, and the interaction term between the independent variable and the moderator was included in the model. Gender was coded as −0.5 = female and 0.5 = male. Conditional effects of childhood traumatic experiences on guilt were examined at the specified values of the moderator. Bootstrapping with 5,000 resamples was used to estimate 95% confidence intervals for the interaction effect. Statistical significance was set at *p* < 0.05.

### 2.6 Ethical aspects

The study was approved by the Ethics Committee of Universidad César Vallejo (Approval No. 0126–2017) (Universidad César Vallejo, 2022), The research adhered to fundamental ethical principles, including autonomy, ensured through voluntary informed consent and assent; confidentiality, by safeguarding participants’ data; justice, by guaranteeing equitable and non-discriminatory treatment; and beneficence, by promoting the potential social value of the research.

In addition, the study complied with international ethical standards for research involving human participants, including the Declaration of Helsinki (World Medical Association, 2024), which emphasizes respect for human dignity, participant well-being, and the minimization of potential risks. The ethical principles outlined by the (American Psychological Association, 2010; 2017) were also followed, particularly those related to informed consent, privacy protection, non-maleficence, and integrity in data collection, analysis, and reporting. Together, these frameworks ensured that the study was conducted with scientific rigor and full respect for participants’ rights throughout all stages of the research process.

## 3 Results

### 3.1 Descriptive data analysis

In Table 1, regarding the distribution and frequency of the childhood traumatic experience variable, it can be observed that the population with traumatic experiences predominates with 96.0% (*n* = 192), followed by the population without traumatic experiences with 4.0% (*n* = 8). That is, 9 out of 10 individuals experience at least one considerably traumatic event.

**Table 1:** Distribution and frequency of the childhood traumatic experience variable.

Level	Childhood Traumatic Experience	
	*n*	%
With traumatic experiences	192	96.0
Without traumatic experiences	8	4.0

*N* = 200. Data extracted using the Child and Adolescent Trauma Screen instrument.

In Table 2, regarding the distribution and frequency of the guilt variable, it can be observed that the moderate level predominates with 42.0%, indicating that guilt is experienced at a level that is neither very intense nor very low (*n* = 84), followed by the low level with 40.5% (*n* = 81). In the shame dimension, the moderate level predominates with 44.5%, meaning that shame is experienced at a level that is neither very intense nor very low (*n* = 89), followed by the low level with 38.0% (*n* = 76). In contrast, in the guilt dimension, the low level predominates with 59.0%, indicating low levels of guilt (*n* = 118), followed by the moderate level with 30.5% (*n* = 61). That is, 3 out of 5 individuals experience guilt.

**Table 2:** Distribution and frequency of the guilt variable.

Level	D1: Shame		D2: Guilt		Guilt	
	*n*	%	*n*	%	*n*	%
High	35	17.5	21	10.5	35	17.5
Medium	89	44.5	61	30.5	84	42.0
Low	76	38.0	118	59.0	81	40.5

*N* = 200. Data extracted using the Guilt and Shame Experience Scale.

According to Table 3, regarding the distribution and frequency of the gender variable, it can be observed that there is no significant difference between the female and male groups in the variables of childhood traumatic experience and guilt, along with their respective dimensions, as *p* > 0.05.

**Table 3:** Distribution and frequency of the gender variable.

Variables and dimensions	Female		Male		*t*	*p*
	*M*	*DE*	*M*	*DE*		
Childhood traumatic experience						
D1: Traumatic experience	4.27	0.24	4.17	0.24	0.28	0.774
D2: PTSD symptoms	18.35	1.23	17.83	1.20	0.30	0.764
D3: General functioning	2.40	0.18	2.27	0.17	0.51	0.606
Guilt	18.67	0.65	18.37	0.58	0.34	0.733
D1: Shame	10.05	0.35	9.97	0.31	0.17	0.865
D2: Guilt	8.62	0.34	8.40	0.32	0.46	0.644

*N* = 200 M = Mean. DE = Standard Derivation. *t* = Student’s *t*-test. p = Significance.

### 3.2 Inferential statistical analysis

In Table 4, corresponding to the female group, it is observed that PTSD symptoms show a moderate positive correlation with guilt (rho = 0.42, *p* < 0.01), indicating that as PTSD symptoms increase, the tendency toward self-blame also increases. Likewise, a moderate positive correlation was found with the guilt dimension (rho = 0.51, *p* < 0.01), suggesting that PTSD symptoms are associated with greater feelings of guilt. Similarly, a weak positive correlation was found with the shame dimension (rho = 0.28, *p* < 0.01), meaning that although the relationship is small, PTSD symptoms are also associated with an increase in the perception of shame. On the other hand, general functioning showed a weak positive correlation with the guilt dimension (rho = 0.28, *p* < 0.01), indicating that lower general functioning is associated with a higher perception of guilt.

**Table 4:** Spearman’s Rho correlation test in the female gender group.

Variables and dimensions	1.1	1.2	1.3	2	2.1	2.2
1.1 Childhood traumatic experience	--					
1.2. PTSD symptoms	0.41**	--				
1.3. General functioning	0.51**	0.58**	--			
2. Guilt	−0.01	0.42**	0.18	--		
2.1. Shame	−0.10	0.28**	0.06	0.93**	--	
2.2. Guilt	0.12	0.51**	0.28**	0.91**	0.73**	--

*N* = 100. **p < 0.01.

In Table 5, it can be observed that there is a weak positive correlation between the childhood traumatic experience variable and the guilt variable (rho = 0.33), which is statistically significant (*p* < 0.01), indicating that greater childhood traumatic experience is associated with higher perceived guilt. Likewise, a weak positive correlation was found between childhood traumatic experience and the shame dimension (rho = 0.26), which is statistically significant (*p* < 0.01). Similarly, a weak positive correlation was found with the guilt dimension (rho = 0.32), also statistically significant (*p* < 0.01). On the other hand, regarding PTSD symptoms and the guilt variable, a moderate positive correlation was found (rho = 0.42), which is statistically significant (*p* < 0.01). In addition, a weak positive correlation was found with the shame dimension (rho = 0.37), statistically significant (*p* < 0.01). Likewise, a weak positive correlation was found with the guilt dimension (rho = 0.39), statistically significant (*p* < 0.01). Similarly, a weak positive correlation was found between the general functioning dimension and the guilt dimension (rho = 0.21), which is statistically significant (*p* < 0.05).

**Table 5:** Spearman’s Rho correlation test in the male gender group.

Variables and dimensions	1.1	1.2	1.3	2	2.1	2.2
1.1 Childhood traumatic experience	--					
1.2. PTSD symptoms	0.40**	--				
1.3. General functioning	0.33**	0.45**	--			
2. Guilt	0.33**	0.42**	0.19	--		
2.1. Shame	0.26**	0.37**	0.12	0.90**	--	
2.2. Guilt	0.32**	0.39**	0.21*	0.92**	0.69**	--

*N* = 100. *p < 0.05, **p < 0.01.

In Table 6, it can be observed that the Childhood Traumatic Experience variable significantly influences guilt (*p* < 0.05). On the other hand, the Gender variable also significantly influences guilt (*p* < 0.05). The Trauma*Gender interaction variable likewise shows a significant influence (*p* < 0.05).

**Table 6:** Table of moderation effect when the outcome variable is guilt.

Model	*B*	se	*t*	*p*	CI 95%	
					LL	UL
Constant	16.87	0.85	19.71	0.000	15.18	18.56
Childhood traumatic experience	0.39	0.17	2.25	0.025	0.04	0.74
Gender	−3.78	1.71	−2.21	0.028	−7.16	−0.41
Trauma*Gender	0.83	0.35	2.38	0.018	0.14	1.52

*N* = 200. B = Coefficient, se = Standard Error, t = Student’s t-value, p = significance, LL = Lower limit, UL = Upper limit.

In relation to Table 7 of the moderation effect, it can be observed that when childhood traumatic experience (X) is considered in the female group (*p* > 0.05), this condition does not significantly influence the variable guilt (Y). On the other hand, when childhood traumatic experience (X) is considered in the male group (*p* < 0.05), it leads to higher levels of guilt.

**Table 7:** Moderation effect table with guilt as the outcome variable.

Gender (W)	*B*	se	*t*	*p*	CI 95%	
					LL	UL
Female (−0.5)	−0.02	0.24	−0.09	0.925	−0.50	0.46
Male (0.5)	0.81	0.25	3.23	0.001	0.31	1.30

*N* = 200. B = Coefficient, se = Standard Error, t = Student’s t-value, p = significance, LL = Lower limit, UL = Upper limit.

The moderation analysis showed that the interaction between childhood traumatic experience (X) and gender (W: female vs. male) was statistically significant, indicating that the relationship between childhood traumatic experience and guilt varies as a function of gender. Specifically, when examining the simple effects, it was found that in the male group the effect of childhood traumatic experience on guilt was positive and significant (*B* = 0.81, *p* < 0.05), with a 95% confidence interval whose lower and upper limits (LL = 0.31; UL = 1.30) were positive and did not include zero. This indicates that, in males, higher levels of childhood traumatic experience are associated with higher levels of guilt. In contrast, in the female group, the effect of childhood traumatic experience on guilt was not significant (*B* = −0.02, *p* > 0.925), as the 95% confidence interval included zero (LL = −0.50; UL = 0.46), indicating the absence of a statistically significant association between childhood traumatic experience and guilt in females. Overall, these findings confirm that gender moderates the relationship between childhood traumatic experience and guilt, such that this positive relationship is present only in the male group and not in the female group.

In addition, the following data were calculated: The interaction between childhood traumatic experiences and gender accounted for an additional proportion of variance in guilt (Δ*R*^2^ = 0.0274), indicating a small but statistically significant moderating effect (*p* = 0.018). These findings suggest that gender contributes meaningfully to the strength of the relationship between trauma and guilt (Figure 1).

**Figure 1:**
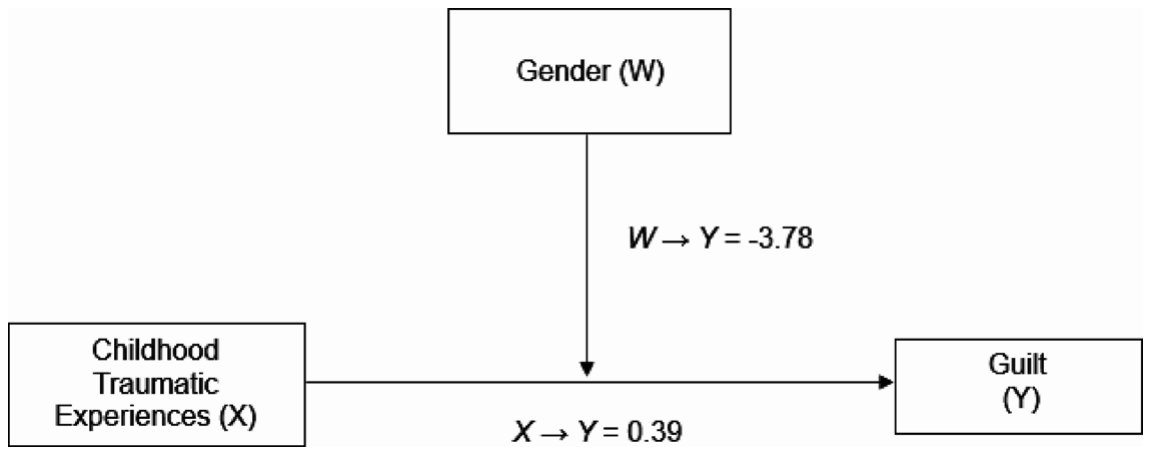
Moderation model of gender in the relationship between childhood traumatic experiences and guilt. X*W → Y = (*B* = 0.83, se = 0.35, CI 95% [LL = 0.14, UL = 1.52]). Male (*B* = 0.81, se = 0.25, CI 95% [LL = 0.31, UL = 1.30]). Female (B = –0.02, se = 0.24, CI 95% [LL = –0.50, UL = 0.46]). This indicates that the strength of the association between childhood traumatic experiences and guilt varies as a function of gender.

## 4 Discussion

The present study examined whether gender moderates the relationship between childhood traumatic experiences and guilt in adolescents. The findings indicate that gender exerts a significant moderating effect, suggesting that the association between childhood trauma and guilt varies as a function of gender. This result is consistent with previous research indicating that exposure to early traumatic experiences is associated with the development of self-conscious emotions such as guilt and shame during adolescence (Cândea and Szentagotai-Tătar, 2018). From the perspective of complex trauma theory (Herman, 1992), prolonged exposure to adverse experiences during childhood may lead to emotional and cognitive alterations that shape how individuals interpret negative events, often promoting feelings of personal responsibility or guilt. While prior studies have reported higher levels of guilt and shame among women (Cândea and Szentagotai-Tătar, 2018), the present findings suggest a different pattern in the strength of the trauma–guilt association, which was significant among male adolescents but not among female adolescents. This divergence may reflect sociocultural differences in emotional socialization, where gender norms influence how emotional responses to trauma are processed and expressed.

The findings further revealed a high prevalence of childhood traumatic experiences, with the vast majority of adolescents reporting exposure to at least one traumatic event. This proportion is notably higher than that reported in previous studies, where 62% of participants indicated at least one traumatic experience (Cândea and Szentagotai-Tătar, 2018). From a developmental trauma perspective, chronic exposure to adverse experiences disrupts neurological and psychosocial functioning (Gregorowski and Seedat, 2013), potentially affecting emotional regulation and social development. These results underscore the widespread nature of trauma during adolescence and reinforce its role as a significant risk factor for later psychological difficulties.

In relation to guilt, moderate levels predominated among adolescents, suggesting that guilt may function as a relatively adaptive emotional response in certain contexts. However, when persistent or excessive, guilt may become maladaptive and contribute to psychological distress. This finding is consistent with previous research indicating that adolescents exposed to traumatic experiences frequently report guilt, which, when intensified, is associated with increased distress and reduced coping capacity (Arcos Naquira, 2019). From a cognitive-emotional perspective, guilt arises from the interpretation of negative events as being linked to one’s own actions or decisions, involving appraisal processes that influence emotional intensity (Kubany and Watson, 2003). Thus, guilt can be understood as an emotion shaped by cognitive interpretations of traumatic experiences, with important implications for adolescent well-being.

With respect to gender differences, no statistically significant differences were found between males and females in levels of childhood trauma and guilt. This suggests that both groups may be similarly exposed to adverse experiences and may develop comparable levels of guilt during adolescence. These findings are consistent with previous research conducted in Peruvian adolescents, where no significant gender differences were identified in experiences of child maltreatment (Baños-Chaparro, 2023). From an attributional perspective, individuals tend to interpret adverse events through processes related to personal responsibility (Janoff-Bulman, 1979). In this context, both male and female adolescents may engage in similar cognitive evaluations of traumatic experiences, leading to the emergence of guilt, even though gender may influence how these emotions are expressed.

The findings also indicate that childhood traumatic experiences were significantly associated with guilt, suggesting that greater exposure to trauma is associated with higher levels of guilt in adolescents. This relationship can be explained from the perspective of moral emotions, where guilt arises when individuals perceive themselves as responsible for negative events or believe they could have acted differently. Attributional theory proposes that individuals tend to attribute adverse events to their own behaviors or characteristics, generating self-blame processes that may intensify when events are perceived as uncontrollable (Janoff-Bulman, 1979). These results are consistent with previous research showing that exposure to multiple traumatic experiences is associated with higher levels of guilt and shame (Stotz et al., 2015). From a cognitive-emotional framework, trauma-related guilt emerges when individuals retrospectively evaluate adverse events and infer personal responsibility, even in the absence of objective control (Kubany and Watson, 2003).

Regarding the influence of gender on guilt, the findings indicate that gender significantly predicts guilt in adolescents, suggesting differences in how self-conscious emotions are experienced across genders. This is consistent with previous research highlighting the role of gender in shaping emotional experiences during adolescence (Roos et al., 2014). These differences may be explained by emotional socialization processes and contextual factors that influence how individuals interpret and regulate their emotions. From an attributional perspective, gender may shape how adolescents assign responsibility to themselves, thereby influencing the intensity and expression of guilt (Janoff-Bulman, 1979).

Importantly, the interaction between childhood traumatic experiences and gender was found to significantly predict guilt, confirming the presence of a moderating effect. Specifically, the relationship between trauma and guilt was significant among males but not among females, indicating that the strength of the association between traumatic experiences and guilt varies according to gender. These findings partially align with previous research identifying gender differences in self-conscious emotions (Lutwak et al., 2001). While prior studies have reported higher levels of guilt and shame among women, the present findings suggest a different pattern in the strength of the trauma–guilt association, which was significant among male adolescents but not among female adolescents. Importantly, this result should not be interpreted as indicating that male adolescents experienced higher overall levels of guilt, since no statistically significant gender difference was observed in guilt scores. Rather, the significant interaction indicates that childhood traumatic experiences were more strongly associated with guilt among male adolescents.

One possible explanation for this pattern involves gendered emotional socialization. During adolescence, socially learned expectations regarding emotional expression, responsibility, and coping may influence how traumatic experiences are cognitively and emotionally processed. Previous research has suggested that gender differences in guilt and shame may be related to socialization and interpersonal sensitivity, while such differences are also influenced by cultural and contextual factors (Roos et al., 2014; Else-Quest et al., 2012). In the Peruvian context, these social expectations may shape how adolescents interpret adverse experiences and respond to feelings of responsibility. Thus, male adolescents may be less likely to openly express emotional distress but may still internalize responsibility-related appraisals following traumatic experiences. This interpretation is consistent with the attributional perspective, according to which guilt may emerge when individuals retrospectively perceive that they could have acted differently or had some responsibility for a negative event (Janoff-Bulman, 1979; Kubany and Watson, 2003). However, this explanation should be considered tentative because emotional socialization, gender-role expectations, coping strategies, and cultural norms were not directly assessed in the present study. Therefore, these factors should be considered potential explanatory mechanisms rather than established explanations of the observed moderation effect.

The Peruvian context may also be relevant to the interpretation of this finding. Gender-related expectations surrounding emotional expression, responsibility, and help-seeking may influence how adolescents respond to and communicate distress following adverse experiences. In this context, the observed pattern may reflect not only individual differences but also culturally shaped expectations about how boys and girls are expected to manage emotional difficulties. However, this interpretation remains tentative because the present study did not directly assess cultural norms, gender-role expectations, or family socialization processes. Future studies conducted in Peruvian adolescent populations could examine these factors directly to determine whether they contribute to differences in the relationship between traumatic experiences and guilt.

Differences in coping styles may represent another potential explanation. Adolescents may differ in how they regulate distress, seek support, avoid emotional disclosure, or cognitively process adverse experiences, which could influence whether traumatic experiences are associated with responsibility-related appraisals and guilt. However, coping strategies were not directly assessed in the present study, and therefore this possibility should be considered a hypothesis rather than an established explanation. Future research could examine whether specific coping strategies contribute to the gender differences observed in the trauma–guilt association.

These results are consistent with the perspective of Else-Quest et al. (2012), who argue that gender differences in emotions such as guilt and shame are generally small and context-dependent. From a developmental perspective, guilt is understood as a moral emotion that emerges through the internalization of social norms and the development of a sense of justice (Villegas de Posada, 1998). In this sense, gender-related expectations may influence how adolescents interpret adverse experiences and assign responsibility, thereby shaping the intensity and expression of guilt.

Additionally, the observed differences may be partially explained by response patterns, as males tended to select more extreme response options, whereas females showed a tendency toward more moderate responses. However, beyond measurement effects, these differences may also reflect gender roles that influence emotional expression. In this regard, male adolescents may be more likely to internalize responsibility in the face of adverse situations, which could contribute to higher levels of guilt when exposed to traumatic experiences.

Although the interaction between childhood traumatic experiences and gender was statistically significant, its incremental contribution to the explained variance was modest (ΔR^2^ = 0.0274). Therefore, the moderating role of gender should be interpreted with caution, as gender accounted for only a small additional proportion of the variance in guilt beyond the main effects of childhood traumatic experiences and gender. The findings should not be interpreted as indicating that gender is a predominant determinant of trauma-related guilt. Rather, gender may represent one contextual factor that influences the strength of this association, alongside individual, family, and sociocultural factors.

### 4.1 Limitations

This study has several limitations that should be considered when interpreting the findings. First, the use of non-probabilistic sampling may have introduced selection bias, limiting the representativeness of the sample. Future studies should consider probabilistic sampling strategies to improve generalizability. Second, the relatively small sample size and the specific characteristics of the study population restrict the extent to which the findings can be generalized to other populations and contexts. Therefore, the findings should be interpreted primarily within the context of Peruvian adolescents with similar characteristics, and future studies with larger and more diverse samples are needed to assess the generalizability of these results. Third, the use of self-report instruments may have introduced biases such as social desirability. Future research is encouraged to incorporate mixed-method approaches that combine self-reports with more objective measures, such as observational techniques or third-party assessments. Finally, other factors that were not assessed in the present study may also influence guilt following childhood traumatic experiences. In particular, family environment, socioeconomic circumstances beyond the socioeconomic criterion used for sample selection, and pre-existing mental health conditions may shape how adolescents interpret and emotionally respond to traumatic experiences. Although adolescents with diagnosed neurological or psychiatric disorders and recent formal psychological or psychiatric treatment were excluded from the study, no comprehensive clinical assessment was conducted to identify undiagnosed psychological or psychiatric conditions. Similarly, other potentially relevant individual, family, clinical, and sociocultural factors, such as intellectual functioning, cultural and educational background, substance use, neurodevelopmental characteristics, family structure, parental psychopathology, social support, and previous mental health experiences, were not directly assessed or statistically controlled for. The absence of these variables from the analysis limits the ability to determine whether they may contribute to the observed association between childhood traumatic experiences and guilt and may therefore result in residual confounding or bias. Future research should consider incorporating these contextual and clinical factors to obtain a more comprehensive understanding of trauma-related guilt during adolescence.

Although cumulative trauma exposure was assessed based on the number of potentially traumatic experiences reported by each participant, the present study did not examine the association between guilt and specific types of traumatic experiences separately. Similarly, the severity, frequency, and chronicity of individual traumatic experiences were not analyzed as separate variables. Therefore, the findings do not establish whether particular types or characteristics of traumatic experiences are more strongly associated with guilt. Future studies should examine specific trauma types and characteristics of exposure, including severity, frequency, chronicity, and cumulative patterns, to determine whether the association between childhood traumatic experiences and guilt differs according to the nature and characteristics of the traumatic experiences. These limitations are particularly relevant when interpreting the observed association, as the present findings should not be interpreted as establishing a causal relationship between childhood traumatic experiences and guilt. Future longitudinal research incorporating more comprehensive assessments of trauma characteristics, clinical and family factors, and relevant sociocultural variables would help clarify the mechanisms underlying this relationship.

### 4.2 Implications

The findings have potential implications for clinical practice, prevention, and public health. Because childhood traumatic experiences were associated with guilt and this association varied according to gender, the results suggest that trauma assessment during adolescence may benefit from considering not only exposure to adverse experiences but also the emotional responses associated with those experiences. In particular, the findings support the potential value of incorporating guilt-related responses into broader trauma-informed assessments, especially when adolescents report a history of traumatic experiences.

From a preventive perspective, the findings may inform school-based programs aimed at promoting emotional awareness, adaptive interpretations of adverse experiences, and early identification of adolescents who may require additional psychological support. Educational and mental health professionals could consider incorporating trauma-informed and gender-responsive approaches when identifying emotional difficulties associated with traumatic experiences. However, the present cross-sectional findings should not be interpreted as demonstrating the effectiveness of any specific screening or intervention strategy. Rather, they provide a basis for future research evaluating whether gender-sensitive assessment and intervention approaches improve the identification and management of trauma-related guilt in adolescents.

At the public health level, early identification of trauma-related emotional responses may facilitate timely referral to appropriate child and adolescent mental health services. The observed moderation effect also suggests that prevention and intervention strategies should avoid assuming that adolescents respond uniformly to traumatic experiences. Nevertheless, gender should be considered one contextual factor among several, rather than a standalone determinant of guilt, given the modest incremental contribution of the interaction effect and the unmeasured individual, family, and sociocultural factors that may influence these outcomes.

## 5 Conclusion

The present study provides evidence that childhood traumatic experiences are significantly associated with guilt among adolescents, and that this relationship varies as a function of gender. Specifically, the findings indicate that while both males and females report similar levels of trauma exposure and guilt, the association between these variables is significant only among male adolescents, highlighting the presence of a moderating effect.

These results contribute to the understanding of trauma-related emotional processes by demonstrating that the link between adverse experiences and self-conscious emotions is not uniform, but rather shaped by gender-related factors. From a psychological perspective, this suggests that the ways in which adolescents interpret and internalize traumatic experiences may differ according to gender, influencing the development and expression of guilt.

Overall, the findings underscore the importance of incorporating gender-sensitive approaches in the study and intervention of trauma-related outcomes during adolescence. Addressing trauma-related guilt, particularly among populations where this association is more pronounced, may contribute to reducing psychological distress and promoting more adaptive emotional processing. In this context, integrating trauma-informed and gender-responsive strategies within mental health and educational settings may enhance the effectiveness of prevention and intervention efforts aimed at improving adolescent well-being.

## Data Availability

The raw data supporting the conclusions of this article will be made available by the authors, without undue reservation.
